# Community Hospital Response to COVID-19 Outbreak

**DOI:** 10.5811/westjem.2021.9.52294

**Published:** 2022-01-17

**Authors:** Nishad Abdul Rahman, Kayla Guidry, Elizabeth Danielle Brining, David Liu, Ngunyi Sandra Leke-Tambo, Adrian Antonio Cotarelo, Miriam Kulkarni, Norman Mok, Raffaele Milizia

**Affiliations:** St. John’s Riverside Hospital, Department of Emergency Medicine, Yonkers, New York

## Abstract

Since early 2020, the world has been living through coronavirus disease 2019 (COVID-19). Westchester County, New York, was one of the hardest and earliest hit places in the United States. Working within a community emergency department amid the rise of a highly infectious disease such as COVID-19 presented many challenges, including appropriate isolation, adequate testing, personnel shortages, supply shortfalls, facility changes, and resource allocation. Here we discuss our process in navigating these complexities, including the practice changes implemented within our institution to counter these unprecedented issues. These adjustments included establishing three outdoor tents to serve as triage areas; creating overflow intensive care units through conversion of areas that had previously served as the ambulatory surgery unit, post-anesthesia care unit, and endoscopy suite; increasing critical care staff to meet unprecedented need; anticipating and adapting to medical supply shortages; and adjusting resident physician roles to meet workflow requirements. By analyzing and improving upon the processes delineated below, our healthcare system should be better prepared for future pandemics.

## BACKGROUND

Not since the 1918 “Spanish” influenza has a pandemic been comparable in clinical severity or transmissibility to coronavirus disease 2019 (COVID-19).^1^,^2^,^3^ There has not been an opportunity in modern literature to record the intricacies of hospital surge planning required to withstand a pandemic of this nature. Early reports from China^4^ and Italy^5^ addressed pandemic surge changes made due to lack of critical care resources. A more recent, American-based model specific to community hospitals, the most common practice setting in the United States,^6^ has not yet been reported.

Westchester County, New York, was the US epicenter of the initial viral surge, with the second greatest rate of COVID-19 cases per capita of any county within the state.^7^ Our hospital, in the county’s largest city Yonkers,^8^ was severely impacted by the pandemic. Our institution is comprised of two acute care hospitals, St. John’s Riverside Hospital – Andrus Pavilion (SJRH-AP) and St. John’s Riverside Hospital – Dobbs Ferry (SJRH-DB) with a total of 150 inpatient beds in a community setting, outside the resources of the larger New York City tertiary care centers. Considering that community hospitals – defined as non-federal, short-term, general hospitals – comprise 85% (5198/6146) of all hospitals in the US,^6^ our experience may provide translatable insights.

We present the challenges that SJRH faced and the key facility changes that were implemented during pandemic surge planning in our Westchester County community hospital as we progressed through our initial COVID-19 pandemic surge from March–July 2020.

### Current Literature

Specific changes made at individual Chinese and Italian hospitals to counteract COVID-19 have been documented.^9^,^10^ However, there has been limited literature published concerning how individual hospitals responded to COVID-19 within the US. Information gleaned from this experience could prove to have utility in the ongoing worldwide battle against COVID-19, as well as potential future infectious disease outbreaks.

### Westchester County COVID-19 Pandemic Impact and St. John’s Riverside Hospital

As of July 1, 2020, the global cumulative confirmed COVID-19 cases totaled 10.6 million with 526,208 deaths. In the United States, >2.6 million cases were confirmed and >127,000 deaths were attributed to COVID-19, with cases continuing to increase at the time of writing.^7^ Within New York State, Rockland County had the highest per capita case rate (4165 cases/100,000 persons), Westchester County had the second highest at 3604/100,000 persons, and New York City had the sixth highest with 2607 cases/100,000 persons.^7^

Our emergency department (ED) has approximately 46,000 annual visits. We are an academic site functioning as a teaching hospital, with two residency programs - emergency medicine and internal medicine. Yonkers is the largest city in Westchester County with 200,000 residents.^8^

## DATA

### Testing

From March 9–July 1, 2020, there were 7791 total ED patients under investigation (PUI) for COVID-19 at SJRH. Of these 7791 PUIs, 829 had positive COVID-19 swabs (10.6%). Notably, due to lack of testing availability, not all PUIs were swabbed for COVID-19, with some cases deemed to be suspicious for COVID-19 clinically by findings such as hypoxia, or by using radiographic signs such as ground-glass opacities on chest radiograph or chest computed tomography.

### Admissions

Of the total 7791 PUIs, 2038 patients were admitted to the hospital (26.2%), 5467 were discharged home from the ED (70.2%) and 2038 were admitted to the hospital. Among the latter, 1178 patients were ultimately discharged from their inpatient hospitalization (1178/2038, 68.2%).

### Mortality

Of the 2,038 PUIs who were admitted to the hospital, 153 died (overall inpatient PUI mortality of 7.5%). Of the total 7,791 PUIs, including those who did not test positive for COVID-19, there were 179 deaths (179/7,791, mortality rate among all PUIs of 2.2%). There were 26 PUIs who died before being admitted to the hospital (26/7,791, 0.3%). Of the 829 PUIs who were found to be COVID-19 positive, 168 patients died (168/829, mortality rate among COVID-19 positive PUIs of 20.2%).

### Transfers

Our mortality data does not include the 222 patients (222/7,791, 2.8%) who were transferred to other facilities. Of those 222 patients, 128 were transferred during their ED visit (57.7%). An additional 94 admitted patients were transferred (94/222, 42.3%).

### Surge Bed Capacity

Over the course of the pandemic, we improved our intensive care unit (ICU) capacity from an initial 12 beds to 55 ICU beds, an increase of over 450% from our usual critical care capacity (55/12, 458.3%). These beds were created using areas that had previously served as the ambulatory surgery unit (ASU), post-anesthesia care unit (PACU), and endoscopy suite (ENDO). Furthermore, we had more ventilated patients than ever, with as many as 65 ventilated patients within our hospital at peak including the ED, PACU, ASU, ENDO, and main ICU.

A total of 130 employees tested positive for COVID-19.

## KEY LESSONS LEARNED

### 1. Establish a screening zone

#### The Tents

In anticipation of a surge in potential COVID-19 patients with unknown acuity, three screening tents were erected outside the ED ambulance bay (Figure). The first two tents were approximately 100 square feet each and were acquired by the hospital. A third, 800 square-foot tent was provided by the Westchester Department of Emergency Management. The large tent became the primary location for intake, medical screening, and discharging stable COVID patients. The original, smaller tents were used predominantly for diagnostic testing - one for radiographs and another for electrocardiograms, parenteral medications, and nebulizer treatments. Combined, these tents established approximately 25 evaluation spaces. Considering the rapid turnaround for most of these patients, this proved sufficient for even large volumes.

In accordance with the federal Emergency Medical Treatment and Labor Act and New York State regulations, our hospital bylaws identify nurse practitioners (NP) and physician assistants (PA) as qualified medical personnel capable of performing a medical screening exam; thus, all patients were screened and triaged to the appropriate treatment area based on illness severity by a NP or PA. To enhance rapid documentation, existing rapid medical evaluation notes were used. Discharge instructions were created and templated to expedite discharge, referrals, and return criteria.

Notably, the exponential surge of COVID-19 within New York and the limited availability of testing kits necessitated that our COVID-19 tests were reserved almost exclusively for patients with high illness severity requiring hospital admission. Per Department of Health testing guidelines at that time, many mildly symptomatic patients without hypoxia or other vital sign changes were evaluated in screening tents and discharged with public health referrals and without COVID-19 testing.

#### The Main ED

Patients with hypoxia, abnormal vital signs, or clinical distress were evaluated in the main ED. Patients who were triaged into the main ED provided their cell phone numbers to staff to be used for registration and history taking, thereby minimizing staff exposures. Emergency medical services (EMS) transporting patients via ambulance called an ED notification for suspected COVID-19 patients prior to arrival and were directed to either the screening tents or into the main ED based on illness severity. All healthcare workers wore personal protective equipment (PPE) within the screening tent and main ED for the entire duration of their clinical shifts.

### 2. Create overflow critical care units. (The ICU is not a place; it is a state of mind.)

Exponentially increasing ICU bed requirements necessitated expanded critical care capacity. All elective surgeries were canceled on March 16, 2020, allowing us to convert areas that had previously served as the ASU, PACU, and ENDO suite into makeshift ICUs staffed by clinicians with critical care training. This included 17 ASU beds, 12 PACU beds, and 12 ENDO beds at SJRH-AP, as well as 12 ASU beds, eight PACU beds, and one ENDO bed at SJRH-DF. Known COVID-19 positive patients often remained within the ED for extended periods, with a significant increase in our average length of stay (LOS) compared to the prior year (11 hours and 20 minutes during the study period compared to 8 hours and 58 minutes in 2019, representing a 26.4% increase in LOS). Thus, the ED came to serve as an adjunct fifth ICU with critical care teams rounding at the bedside.

Upon evaluation of our heating, ventilation and air conditioning (HVAC) systems, we discovered that the ASU and PACU units were part of a non-ducted system, which does not permit isolated air transport to individual rooms as a ducted system would. Individual, non-ducted units were fitted with portable, high-efficiency particulate air filters. Limited space led to admitted COVID-19 positive patients requiring ventilation being grouped into shared rooms, with as many as four patients to a room. Moving forward, our institution will be converting a non-telemetry floor to a ducted HVAC system with increased electricity supplies for ventilator use. This will serve as the ICU overflow unit for future surges that may similarly require extended ICU-level admissions.

We recommend that hospital systems prioritize and install ducted systems to facilitate rapid conversion into isolation rooms in the event of a pandemic surge.

### 3. Increase critical care staffing corresponding with patient volume

The starkest personnel shortage we faced was the unprecedented need for critical care-trained nursing staff. We trained non-critical care, in-house nurses and hired a total of 41 travel nurses to meet the needs of the ED and ICUs, supplementing the previous 47 ED nurses and 29 ICU nurses. The nursing department provided courses on critical care medication and ventilator management to nurses with minimal prior ICU experience. Nursing supervisors reassigned nurses with ICU experience from other departments to staff the surge ICUs, forming teams composed of non-critical care nurses working under the supervision of a nurse with formal ICU training and experience.

New York’s rigorous standards for medical clearances, licensing, credentialing, and hiring protocols challenged our institution’s ability to quickly transition travel nurses onto the floors. Unfortunately, our first travel nurse was unable to work clinically until April 9, 2020. By comparison, the highest daily confirmed positive COVID-19 cases we had at SJRH occurred on April 4. This limited the capacity of travel nurses to intervene during the steepest phase of COVID-19 case growth. Furthermore, we experienced the paradoxical issue of ED understaffing during the pandemic surge and overstaffing after ED volume had drastically decreased. Effective allocation of surge staffing requires a protean mindset and a culture of adaptability.

We suggest the early inception of credentialing for short-term critical care-trained staff. Anticipate increased nursing and physician need in the ED and the ICUs early in the surge, with decreased ED staffing needs later. Allow flexible allocation of critical care staff, as ED volume may increase exponentially and then rapidly plummet, while inpatient ICUs remain full for weeks beyond the peak of the surge.

### 4. Implement the Use of Reusable Personal Protective Equipment Gowns

St. John’s Riverside Hospital used up its entire supply of disposable gowns within the first week of the pandemic surge. This issue was compounded by a national shortage of disposable gowns in late April 2020, leading to prices for disposable gowns increasing by 300%. To address gown shortages, our hospital transitioned to purchasing washable PPE to meet standard PPE requirements. The shortage of disposable gowns continued through the peak of the surge, and coveralls or “bunny suits” were the most prominent form of PPE used during the majority of the surge.

To control and prevent further spread of the COVID-19, we advise changing to reusable PPE and investing in coveralls or “bunny suits” early on. Alternatively, any system to ensure adequate disposable PPE must be ready to counter massive surges in need and potential nationwide shortages.

### 5. Expect Medical Supply Shortages

Supply challenges were an inextricable complication of the pandemic. Conversations by leadership through the Greater New York hospital network helped facilitate movement of patients and equipment between hospital systems, and daily calls within the SJRH network were critical in combatting shortages. On the upslope of the pandemic surge curve, there was a national shortage of disposable gowns, D5W intravenous (IV) fluids, and small N-95 masks. Other specific shortages included the following:

- Central line kits
- Ventilators and ventilator circuits
- Arterial blood gas (ABG) kits
- Rigid stylets
- Endotracheal tube holders
- Feeding tubes
- Yankauer suction tubes
- Fentanyl
- Bougies
- Sedation medication such as propofol and midazolam
- Vasopressors such as norepinephrine, vasopressin, and epinephrine
- Gloves
- Disinfectant wipes
- Ultrasound probe covers.

Additionally, we recommend flexibility and innovation to counteract temporary shortages. For example, pseudo-ABG kits were created and used by drawing heparin into 5-cc syringes. For short periods, long 14G IVs were placed in lieu of traditional triple lumen central lines to facilitate centrally acting medications such as pressors. This temporizing measure allowed the bridging of patients until resupply.

We recommend that hospitals stockpile critical medical supplies and work aggressively to establish adequate supply chains in partnership with neighboring healthcare organizations.

### 6. Resident Physician Roles

Resident physicians played a critical role in our hospital’s response to this pandemic. With many hospitals hosting one or more residency programs, it is important to discuss the optimal utilization of these staff members. Specifically, it is essential to assign resident physicians to roles that are consistent with their training and with hospital staffing needs.

All electives were canceled during the surge, and residents worked exclusively within either the ED or one of the COVID ICUs. Cancelled electives for emergency medicine residents included ultrasound, neonatal ICU, neurosurgical ICU, EMS, and anesthesia. Cancelled rotations for internal medicine residents included gastroenterology, geriatrics, nephrology, and outpatient medicine. Eight of 30 emergency medicine residents and seven of 30 internal medicine residents were pulled from their electives. Senior residents were responsible for rounding on all ventilated patients within the ED, ensuring continuity of care and adjustment of medications and ventilator settings as needed. Residents were of particular utility within COVID ICUs, especially overnight, with critical care attendings available by phone. To ensure wellness, shifts were restricted to 12 hours, with a dedicated day team and night team for ICU care.

We advocate that resident physicians be integrated into ED and ICU care in a manner that optimizes patient care, educational opportunity, and resident wellness.

## CONCLUSION

The last pandemic surge of this severity and clinical acuity was over 100 years ago, long before the development of modern medicine, including intensive care units and ventilators. To our knowledge, there are no current reports that address the changes necessary for a US community hospital experiencing a pandemic surge. Our institution’s experience through the pandemic as a community site at a major national epicenter provides a vital perspective on the future of pandemic response, as well as the present COVID-19 crisis.

We advise community institutions to increase critical care staffing in accordance with patient volume, create overflow critical care units, evaluate their HVAC systems, establish screening zones, use available resident physicians in the ED and ICUs, and secure supply chain for PPE and critical care supplies.

## Figures and Tables

**Figure:**
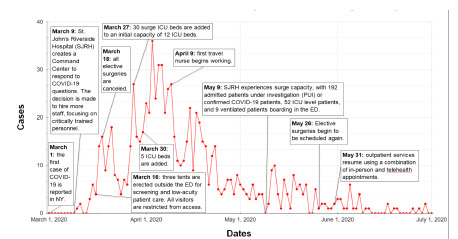
Timeline of key pandemic events compared with Saint John’s Riverside Hospital confirmed COVID-19 cases (March 1, 2020–July 1, 2020). *ICU*, intensive care unit; *COVID-19*, coronavirus disease 2019; *NY*, New York; *ED*, emergency department.
